# Universal association between depressive symptoms and social-network structures in the workplace

**DOI:** 10.1038/s41598-022-14366-9

**Published:** 2022-06-17

**Authors:** Jong-Hyeok Lee, Nobuo Sato, Kazuo Yano, Yoshihiro Miyake

**Affiliations:** 1 Department of Computer Science, Tokyo Institute of Technology, Yokohama, Kanagawa 226-8502 Japan; 2 Happiness Planet, Ltd., Kokubunji, Tokyo 185-8601 Japan; 3 Hitachi, Ltd., Kokubunji, Tokyo 185-8601 Japan

**Keywords:** Quality of life, Human behaviour, Occupational health

## Abstract

An unhealthy communication structure at a workplace can adversely affect the mental health of employees. However, little is known about the relationship between communication structures in the workplace and the mental health of employees. Here, we evaluated the face-to-face interaction network among employees (N = 449) in a variety of real-world working environments by using wearable devices and investigated the relationship between social network characteristics and depressive symptoms. We found that the cohesive interaction structure surrounding each individual was negatively correlated with depressive symptoms: a universal relationship regardless of occupation type. This correlation was evident at the group scale and was strongly related to active interactions with abundant body movement. Our findings provide a quantitative and collective perspective on taking a systematic approach to workplace depression, and they suggest that the mental health of employees needs to be addressed systematically, not only individually.

## Introduction

Humans are social animals that organize and interact with each other. The workplace, which contributes to the productivity of society, represents where modern people spend most of their time. At the same time, stress arising from the workplace is a major cause of depression among people^1,2^. Depression not only leads to the deterioration of an individual’s mental and physical health, but it also impairs their ability to function in society, causing huge losses in productivity. This disorder has become a major problem in modern society^3–6^.

Various studies have investigated psychological and pharmaceutical interventions for depression and their effects in the workplace^7–10^, but most of the interventions have relied on conventional methods focused on the individual, which implicitly suggests that external factors are difficult to change. However, the social factors underlying depression in the workplace are fundamental and prevalent. In the workplace, employees may be exposed to various problems such as bullying, harassment, poor organizational climate, difficult relationships, and high demands, for which they have little control over and receive low support for coping with^11–14^. These social factors are closely related to how people interact. In an organization, interaction takes place not only between individuals but also collectively, in which contexts such as isolation, close-knit group formation, and information flow are formed. Since these collective contexts are difficult for individuals to deal with on their own, it is necessary to systematically reduce the burden of individual employees through expanding the understanding of social interactions within organizations related to depressive symptoms, and analyzing and managing them in a quantitative and continuous way. One means of meeting these needs is to observe the social network. Social network analysis has been used as a quantitative methodology to explain social phenomena within fields ranging from psychology to economics^15^. With direct empirical findings that the social network structure is related to the brain activity during social interaction^16,17^, there has recently been an increasing demand for a comprehensive approach to mental health that takes into account a person’s social network^18–20^. Understanding the interactions within social networks associated with depression in the workplace could open new opportunities for fundamental approaches to prevention and diagnosis of depression and to interventions beyond the conventional individual approach.

There is a prominent study demonstrated that depressive symptoms are not just individual symptoms, but social symptoms related to people around them^21^. This study showed that depressive symptoms tend to spread to the surroundings on the social network constructed through friends and neighborhood relationships. However, the phenomenon focused on in this study deals with the change in depressive symptoms that has occurred in social networks constructed based on the ‘relationships’ such as friends or neighbors over a long period of 20 years. Therefore, this study did not directly deal with the various social contexts formed from the social networks, such as the frequency of interactions or the density of interactions or the centrality, that are actually happening around us in real life. And indeed, the results of this study were not significant among co-workers, showing that the ‘relationship’ defined only according to the place of employment may not be sufficient to observe a meaningful social impact. To accurately understand workplace interactions, it is necessary to observe the actual interactions of employees. Among the various interactions, face-to-face interaction is a communication channel whereby verbal information as well as nonverbal information and emotional expressions are exchanged^22–24^. Such interaction plays an important role in the formation of an efficient knowledge worker team^25^ and coordination of conflict in an organization^26^. Recently, wearable sensor technology enabling detecting face-to-face interaction, along with a related social network analysis method, has been proposed for quantitative observations in organizations^27–32^. These studies not only allow investigating the real interactions within the organization, but also suggest that the characteristics of the social network constructed of the interactions could be factors that can estimate the process of information exchange, role, productivity, relationship, and circumstance within the organization. However, few studies have investigated the social network characteristics directly related to depressive symptoms, and no study has investigated a variety of real working environments in a consistent method.

**Figure 1:**
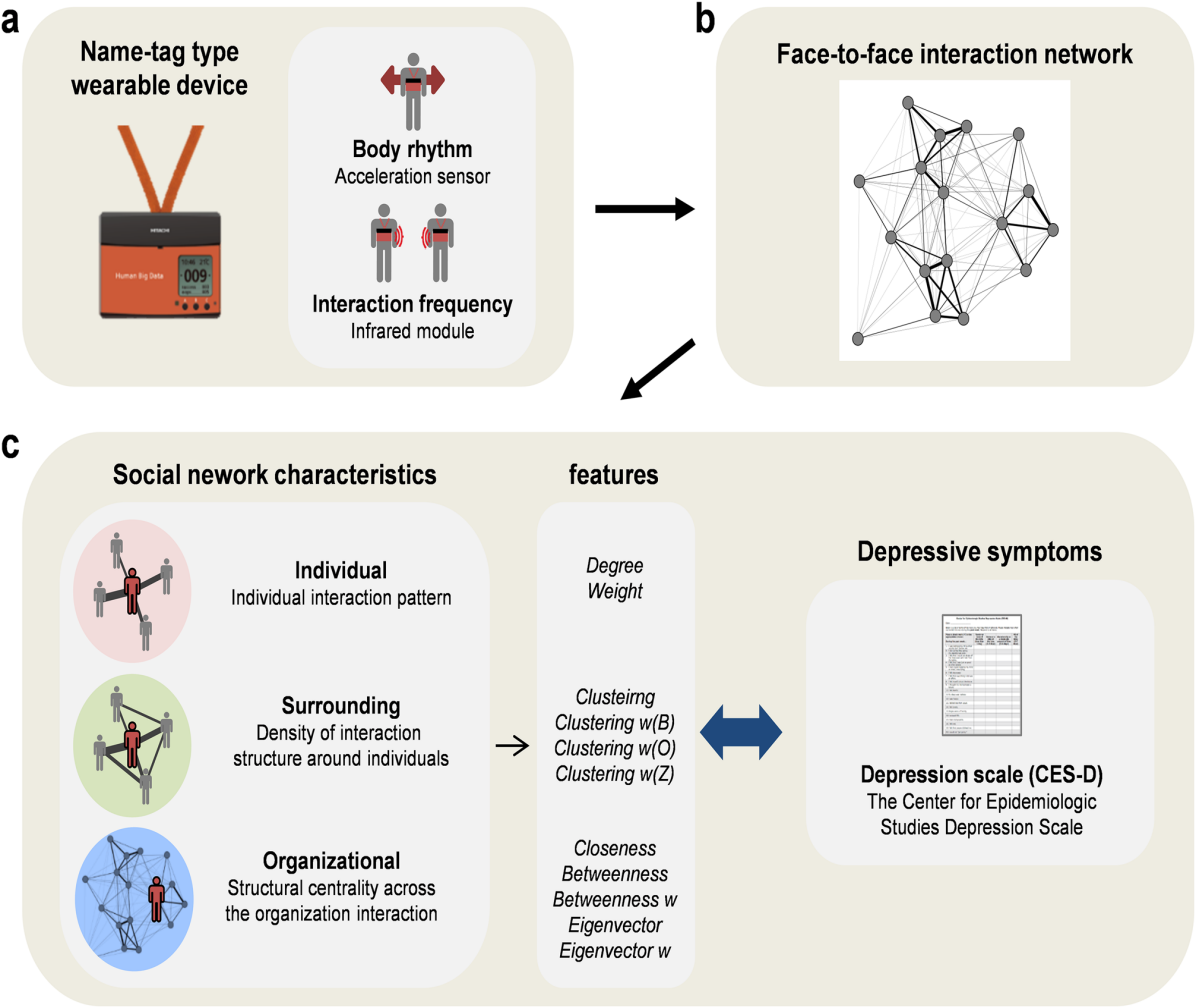
Analysis procedure. (**a**), A wearable name-tag-type device detected body movements and interactions. Body movements were measured from the zero cross count of the acceleration near the chest, and interactions were measured through infrared module detection with other devices. (**b**), The workplace’s face-to-face interaction network was constructed from the interaction information. The average number of interactions per day was used to weight each link. Body movements were used to estimate the degree of activity of each interaction and to compare the characteristics of networks composed of active and inactive interactions. (**c**), The social network characteristics extracted from various scales of the face-to-face interaction network were compared with the depressive symptoms assessed by the depression scale (CES-D). See “Methods” section for detailed description of social network characteristics and depressive symptoms.

In this study, we aimed to investigate the universal relationship between social network characteristics and depressive symptoms in the workplace. To capture the universality of the relationships, which are not limited to a specific workplace, we investigated 449 people from 10 organizations in various occupations. To measure the interaction, we used a device that is similar to a name-tag (Fig. 1a) and designed to be comfortable in a work environment^33^. This wearable device can detect face-to-face interactions and body movements during the interactions. All interaction data were recorded for more than 5 consecutive days to capture chronic interaction features. For detailed data descriptions, please see “Methods” section. We used the face-to-face interaction information to create a network structure (Fig. 1b) and quantified not only an individual’s interaction characteristics but also the contextual characteristics of the surrounding interaction density and centrality across the entire organizational interaction (Fig. 1c). To evaluate depressive symptoms, we used a self-reported depression scale (Center for Epidemiologic Studies Depression scale; CES-D^34^), which has been widely used in the screening and treatment of depression and in epidemiological investigations in the general population. We analysed the relationship between social network characteristics and depressive symptoms in all organizations and within each organization to investigate the universality of the relationship. In addition, we examined the structure and scope of the social network characteristics related to depressive symptoms. Furthermore, we distinguished the activeness of interactions based on body movements during the interactions, and compared the social network characteristics related to depressive symptoms in each network composed of active and inactive interactions. Through these processes, we explore the characteristics of social networks that have a significant relationship with depressive symptoms within the organization and their contextual meanings.

This study examined a variety of workplaces in a consistent method to determine the relationship between depressive symptoms and interaction features. The results are meaningful because they provide universal evidence for workplace depression. Our results also indicate that the density of the surrounding interactions has a negative relationship with depressive symptoms, and they provide a new perspective on a systematic approach to workplace depression.

## Results

### Correlation between social network characteristics and depressive symptoms

**Figure 2:**
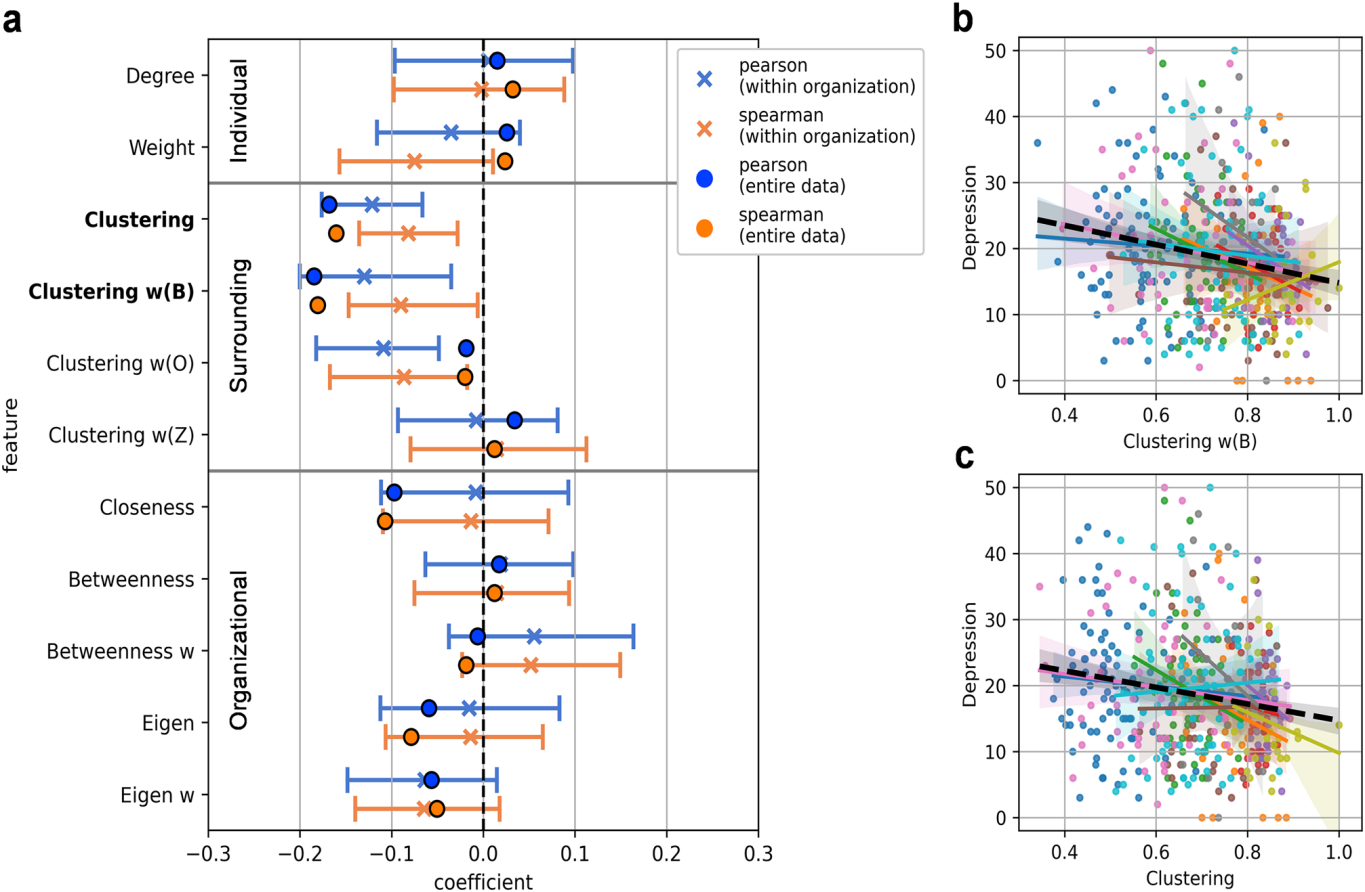
Correlation between social network characteristics and depression score. (**a**) The circle-marker indicates correlation coefficients for the entire dataset (N = 449). The x-marker and error bar indicate the mean and 95% confidence interval of correlation coefficients within each organization (N = 10) calculated by 1000 bootstrap samples. Features in bold type have significant correlations in both Pearson and Spearman correlations in the entire dataset (*p* < 0.05; two-tailed *p* value adjusted by Benjamini–Hochberg method). (**b**), (**c**), Each scatter plot shows the relationship between *Clustering w(B)*, *Clustering*, and depression score. The black dashed line indicates the linear regression of the entire dataset, and each coloured solid line indicates the linear regression within a single organization.

From social networks composed of face-to-face interactions observed via wearable sensors, we extracted social network characteristics at the individual scale, the surrounding scale, and the organizational scale (Fig. 1c; details are explained in Methods). We investigated the correlation between social network characteristics and depression score across the entire dataset (Fig. 2a ‘circle’ marker) and within each workplace (Fig. 2a ‘x’ marker) to ascertain the universality of the relationships. For a comprehensive analysis including nonlinearity of features, we investigated the Pearson (Fig. 2a, blue) and Spearman (Fig. 2a, orange) correlation coefficients between social network characteristics and depression score.

In the analysis of the entire dataset, *Clustering* and *Clustering w(B)*, defined according to the surrounding scale, showed a significant negative correlation with depression score (*Clustering*: Pearson’s r = − 0.16 [*p* = 0.002], Spearman’s r = − 0.16 [p = 0.004]; *Clustering w(B)*: Pearson’s r = − 0.18 [*p* < 0.001], Spearman’s r = − 0.18 [*p* = 0.001]; two-tailed p-value adjusted by Benjamini–Hochberg method). These relationships also tended to have a negative correlation within each workplace (*Clustering*: Pearson’s r = − 0.12 [− 0.18, − 0.06], Spearman’s r = − 0.08 [− 0.13, − 0.03]; *Clustering w(B)*: Pearson’s r = − 0.13 [− 0.20, − 0.04], Spearman’s r = − 0.09 [− 0.15, − 0.00]; The square brackets indicate 95% confidence intervals). In Fig. 2b,c it can be seen that *Clustering w(B)* and *Clustering* show a negative relationship with depression score in entire data (black dot line) and in most organizations (coloured solid line).

The clustering coefficients have a higher value as there are more interactions between an employee's interaction partners. Therefore, a high clustering coefficient indicates that the employee is more likely to belong to a close-knit group of face-to-face interactions. Among the social network characteristics defined at various scales, the prominent result in the clustering coefficients indicates that the depressive symptoms are best explained when the observation scale of the social network characteristics centres on the surrounding people, rather than the individual or the entire organization. The negative correlations across the entire dataset and within each organization suggest that it is a universal relationship in the workplace regardless of occupation.

Differences in weighted clustering coefficients provide additional information about the results. When the frequency of interaction was weighted around an individual, *Clustering w(B)* emphasized the direct interaction frequency between an individual and surrounding people, while *Clustering w(Z)* emphasized the indirect interaction frequency among an individual’s surrounding people (*Clustering w(O)* used both direct and indirect interaction; see “Methods” section for details). Unlike *Clustering w(B)*, no significant correlation was observed in *Clustering w(Z)*. These findings suggest that an employee’s low levels of depressive symptoms are associated with the formation of dense surrounding interactions centred on the people with whom the employee interacts frequently, which means the employee is actively participating in such interactions and not simply having frequent surrounding interactions.

### Structural and scale nature of the relationship between *Clustering w(B)* and depressive symptoms

**Figure 3:**
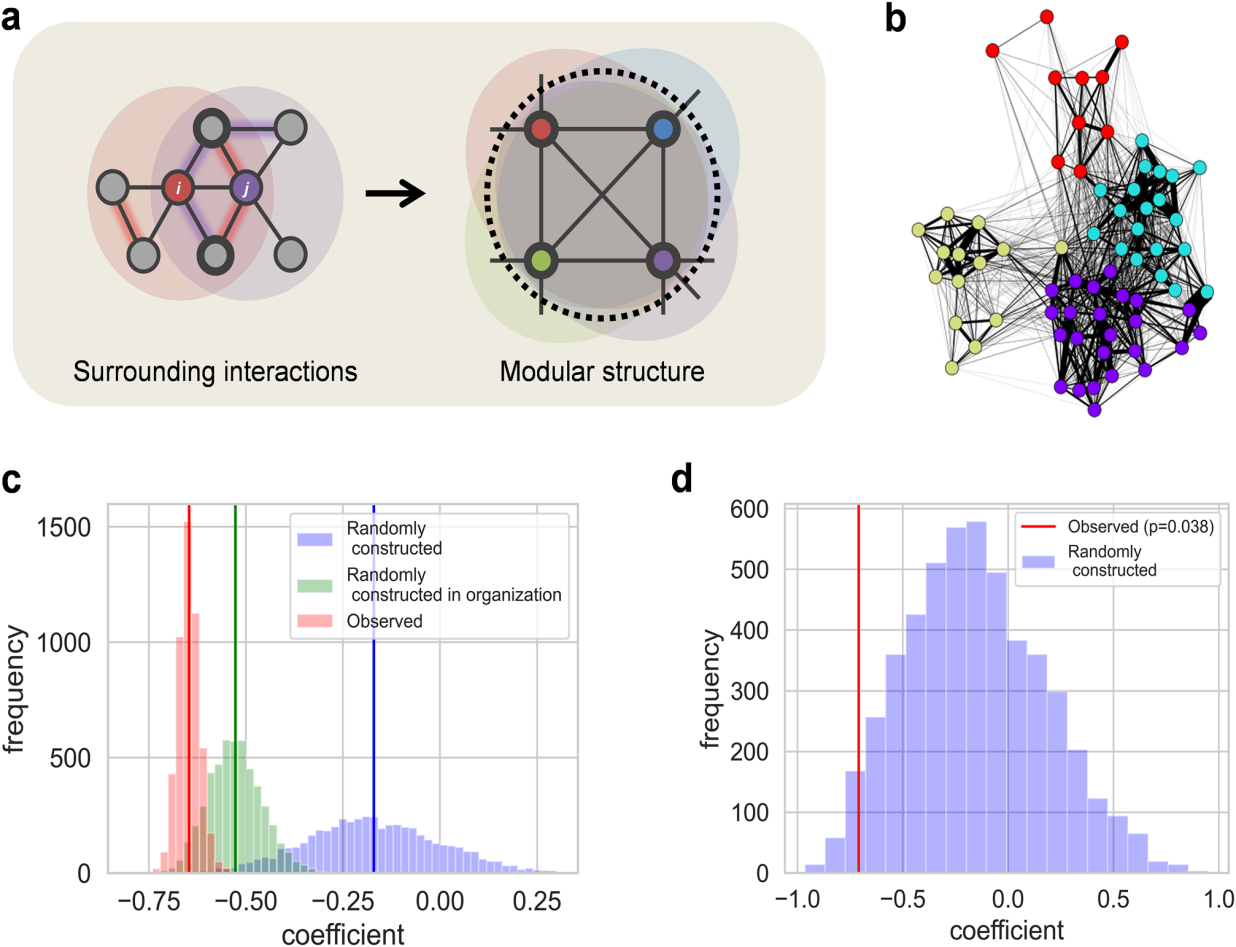
Modular group structure of the face-to-face interaction network and the relationship between *Clustering w(B)* and depression score in the group unit. (**a**) (Left) Surrounding interaction overlaps by the number of neighbours (bold lines) shared with neighbouring nodes. (Right) In a modular network structure, since each node’s surroundings overlap at a high rate within the structure, this structural unit significantly influences the clustering of the nodes belonging to it. (**b**) A representative visualization of the face-to-face interaction network of one workplace. The width of the link indicates the frequency of interaction, and the colour of the node indicates the community constructed by the community detection algorithm. Modular community structures are observed where frequent interactions occur internally. (**c**) The distributions of the Spearman correlation coefficient between the average *Clustering w(B)* and the average depression score of each community are shown. Each distribution represents the correlation coefficient of the community structures observed by the community detection algorithm (Red), randomly constructed (Blue), and randomly constructed within each organization (Green). Since the Louvain algorithm^36^ used for community detection led to some variation in the results depending on the order of randomization in the extraction process, the coefficients in the observed community are also represented as a distribution. Each distribution was constructed through 5000 random sampling results. (**d**) The Spearman correlation coefficients between the average *Clustering w(B)* and the average depression score of each organization (Red) and each randomly constructed organization (Blue; 5000 random sampling) are shown.

The clustering coefficient is not independent for each individual in that it depends on the mesoscale network structure defined by the density of interactions among surrounding people; therefore, it is partially shared with surrounding people. For example, in the neighbouring nodes *i* and *j* in Fig. 3a, surrounding interactions formed through the nodes’ common neighbour node (indicated by a bold line in Fig. 3a) are included in their respective clustering coefficients. This property of the clustering coefficient is more prominent in a compact and modular network structure. In a modular network structure in which the internal interactions are sufficiently dense compared with the external interactions, a large proportion of the surrounding interactions of each node are formed through the people within this structure (Fig. 3a). This mesoscale nature of the clustering coefficient allows extending the scale of the clustering coefficient from the individual scale to the group scale in a modular network structure.

A modular network structure called community structure is a prominent structural characteristic in social networks. In the face-to-face interaction networks observed in 10 workplaces, the modularity^35^, a measure of the proportion of interactions within communities, was 0.42 (SD = 0.10; min 0.18 to max 0.62), meaning that there were community structures (Fig. 3b) with a higher density of internal interactions than in a random state (see “Methods” section for detail). Therefore, we examined whether the negative correlation between *Clustering w(B)* and depression score which showed the strongest correlation at the individual scale (see Supplementary note 1 for results of other features) could be significantly extended to the group scale of modular community. To confirm the significance, we compared the correlation between the average *Clustering w(B)* and the average depression score of observed modular communities with the correlation seen in the randomly constructed communities. The modular communities were detected by the Louvain algorithm^36^, a widely used community detection algorithm, and randomly constructed communities had the same number of members as the observed community. Each sampling was performed 5000 times, and the Spearman correlation coefficient was used for robust analysis of the influence of outliers.

The correlation between *Clustering w(B)* and the depression score (Fig. 3c red; − 0.65, SD = 0.03) in observed communities showed a stronger negative correlation than the correlations in the randomly constructed community (Fig. 3c blue; − 0.17, SD = 0.17). In addition, the correlation in observed communities was also higher than that in randomly constructed communities within each organization (Fig. 3c green; − 0.53, SD = 0.07). This means that the community structure as a unit can effectively explain the correlation between *Clustering w(B)* and depression score. Meanwhile, the randomly constructed community within the organization (Fig. 3c green) showed a stronger negative correlation than the randomly constructed community from the entire dataset (Fig. 3c blue), indicating that there is also a difference in the trends of *Clustering w(B)* and depression score throughout each organization. Given that the correlation between *Clustering w(B)* and the depression score in the organization unit (Fig. 3d red) was significantly lower (Spearman’s r = − 0.71 , one-tailed *p* = 0.038) than that in a randomly combined organization unit (Fig. 3d blue) indicates that the organization can also be a valid observation unit.

These results show that the relationship between *Clustering w(B)* and the depression score was not simply due to individual characteristics, but was a group-scale phenomenon related to the modular group structure composed of interactions. At a group level, a centralized and sparse interaction structure reduced the clustering of members. This outcome suggests that employees show low depressive symptoms in a group with flat and dense interactions (e.g., fully connected network) rather than hierarchical and sparse interactions (e.g., star-shaped network).

### Active and inactive interaction in the relationship between clustering coefficients and depressive symptoms

**Figure 4:**
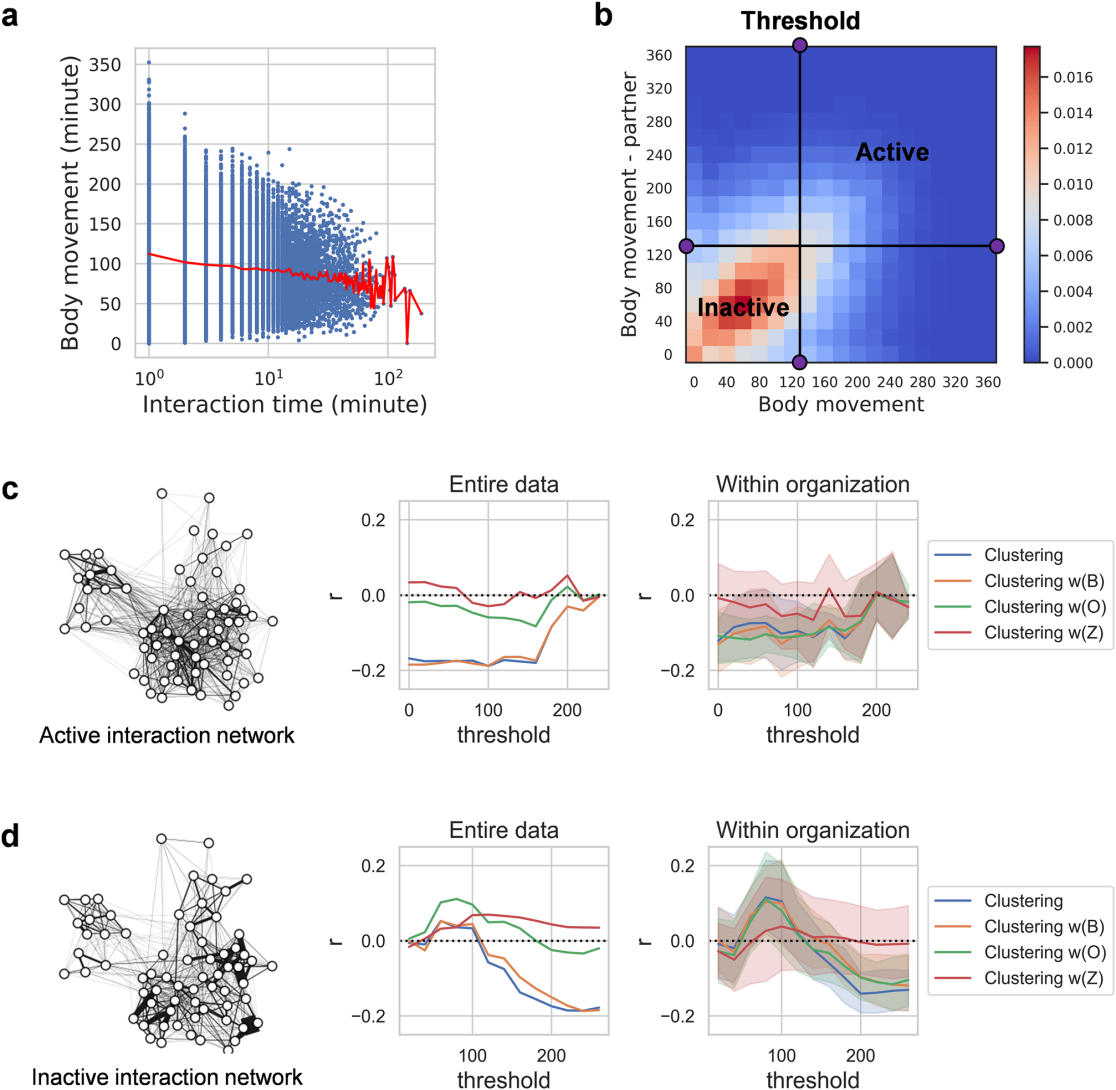
Importance of active and inactive interactions in the relationship between clustering and depressive symptoms. (**a**) The graph shows the relationship between the interaction time and the average body movement of the people who participated in the interaction. The red line is the average body movement for each interaction time, and it shows that the average body movement of people tended to decrease as the interaction time increased. (**b**) The average body movement distribution of individuals and counterparts in all interactions. The colour indicates the density of the interactions. For a specific body movement threshold, if both individuals show a high level of movement, it is an active interaction, and if both show low-level movement, it is an inactive interaction. (**c**), (**d**), The figures on the left side of (**c**) and (**d**) show the structural differences between active and inactive interaction networks at a threshold of 100. Changing the threshold caused changes in the Pearson’s correlation between clustering coefficients and depression scores in the network composed of active interaction (**c**) and network composed of inactive interaction (**d**). The figure on the middle shows the correlation for the entire dataset, and the figure on the right shows the correlation within each organization (band means 95% confidence interval of 1000 bootstrap samples).

Body movements during an interaction can reveal non-verbal interactions such as nodding of the head or using hand gestures and body positioning which are important indicators of emotional expression^22,24^. Therefore, such movement can be helpful for understanding the quality of interaction contextually (e.g., formal/informal situation). In face-to-face interactions observed at the workplace, the average body movements of employees tended to decrease as the interaction time increased (Fig. 4a), and the body movements of the interacting employees showed significant correlation with each other (Fig. 4b; Pearson’s r = 0.28, two-tailed *p* < 0.001, N = 47,768). These characteristics of the measured body movements indicated that non-verbal interactions, which could be reduced in long formal situations such as work meetings, were reflected in the measurements and such contextual background was shared with interaction partners.

Based on this understanding, we classified an interaction as active if body movements of two interacting people were higher than the threshold and as inactive if body movements of two interacting people were lower than the threshold (Fig. 4b). By changing the threshold from a small value to a large value, we observed a change in the Pearson’s correlation coefficient between clustering coefficients and depression scores in the networks composed of active interactions (Fig. 4c) and inactive interactions (Fig. 4d). Both the correlations in the entire dataset and within each organization were investigated. *Clustering* and *Clustering w(B)* maintained a stable negative relationship for both the complete dataset and each organization until inactive interactions (< 160) were removed (7.78% of the total interactions). In contrast, when active interactions (≥ 160) were removed (66.77% of the total interactions), the negative relationship began to disappear, and even the correlation coefficient within each organization appeared as a positive value when active interactions ≥ (100) were removed (31.71% of the total interactions). This outcome shows a high contribution of active interactions to the negative relationship between clustering coefficients and depression scores. Compared with other clustering coefficients, *Clustering w(Z)* showed a weak trend both in overall data and within individual organizations, suggesting again the high contribution of interactions between people with a high frequency of direct interaction. *Clustering w(O)* showed almost the same pattern as *Clustering* and *Clustering w(B)* within each organization, but the level decreased in the overall data because of a bias in the interaction frequency at each organization. For this reason, among weighted clustering coefficients *Clustering w(B)*, which normalized the weight of clustering at each individual level (see “Methods” section for details), had an advantage in quantifying the universal characteristics of the surrounding interaction density.

Taken together, the negative correlation between depressive symptoms and the clustering coefficient was largely due to the clustering of active interactions with abundant body movements.

## Discussion

Various studies^15,16,21,32^ have shown how much social interaction and relationships are related to mental health, but a universal consensus is lacking on quantitatively measurable interaction patterns related to depressive symptoms. Here, we specify the social network characteristics universally related to depressive symptoms in face-to-face interaction networks in workplaces including a variety of occupations. First, we found that clustering coefficients, which indicate the density of surrounding interactions, were negatively related to depressive symptoms. In particular, a stronger relationship was shown when the interaction density was high around people who interacted frequently: *Clustering w(B)*. Second, we confirmed that the relation between *Clustering w(B)* and depression score was distinct in the analysis of community and organization unit, indicating that *Clustering w(B)* was not just a characteristic of one person, but a group-scale phenomenon in which people interact densely. Finally, we found that this relationship was evident in the network composed of active interactions with abundant body movements, but not in the network composed of inactive interactions, which suggests that contextual factors such as formal and informal situations may be involved in this relationship.

The finding that the social network characteristics related to depressive symptoms could be confirmed on the ‘surrounding’ scale provides new perspectives on the understanding of workplace depression. It suggests that the conventional approach of monitoring stress level by using individual’s physiological and social signals^37,38^ could be complemented by expanding the observation to a group scale that includes interactions, when applied to the actual workplace. It also suggests that workplace depression needs to be understood differently from centrality indicators^31,39–44^, which have attracted attention as a method of assessing influence based on the flow of information. The importance of the surrounding interaction density in social networks has been discussed from various perspectives. Such density has been reported to be associated with high productivity^45,46^, academic achievement^47^, prosocial behavior^48^, and brain activity in response to social exclusion^16^. Some studies have also emphasized the importance of interaction and its activeness in informal situations such as resting time^46,47,49^. Taken together with our findings, the evidence suggests that a group’s functionality and organizational members’ psychological health could be matched in the same way.

There are several limitations to the interpretation of our study. First, our results do not contain personal information such as age and gender, and their interpretation is therefore limited. However, the findings across 10 organizations in different occupations suggest a universal relationship, and they support the validity of our interpretation in a variety of settings. Second, the reliability and the interpretation of causality are limited because our results do not consider changes in a social network structure and depressive symptoms. Nevertheless, it can be expected that there will be no significant change within our observation period because the CES-D, the depressive symptom scale we use, showed adequate test–retest reliability over a period of 2 weeks or more in various studies^34,50–53^. Also, an analysis in which a social network was sampled by N consecutive days confirmed a stable negative correlation with the depressive symptoms in the interaction data observed over about 3 consecutive days (Supplementary note 2). Third, our findings emphasize the universal association between depressive symptoms and social network structures, but the interpretation of ‘universality’ is limited in that it is a result from 10 Japanese companies. Future studies can expand the understanding of the universality or specificity according to cultural contexts by investigating numerous organizations in various cultures. Fourth, our main analysis focused on the sum of depressive symptoms (CES-D). This approach implicitly assumes that depressive symptoms are generated by an underlying disease entity. However, researchers in psychometrics and psychopathology have argued that depressive symptoms do not simply appear as an effect of an underlying disorder, but that depression should be understood as a system in which the symptoms are causally connected^54,55^. From this point of view, we should not only consider the relationship of social network characteristics with a total score of depression symptoms but also with individual depression symptoms. In Supplementary Note 3, we investigated the correlation between each item of the CES-D scale and social network characteristics, and found that *Clustering*, *Clustering w(B)* showed an overall negative correlation with the items of CES-D, but showed a relatively strong negative correlation with the items related to somatic symptoms: e.g. “I felt that everything I did was an effort”, “My sleep was restless”. Focusing on individual depressive symptoms might be more insightful for understanding depression within the organization. For example, sleep disturbances are associated with psychosocial factors such as interpersonal conflict, social support, fairness, and social cohesion^56–61^, and are known to have bidirectional associations with depression^62,63^. The link between the cohesiveness of interactions and individual’s somatic symptoms including sleep disturbances might be related to the mechanism of depression within the organization. Alternatively, the relatively prominent characteristics of somatic symptoms might be due to the cultural influences that Asians tend to emphasize somatic symptoms of depression compared to Westerners^64–67^. Investigating these relationships in detail could help to understand depression within the organization and enable effective interventions. Finally, the causal interpretation of the correlation between clustering and depressive symptoms is limited. There are several possible causal interpretations of this correlation: (1) Interaction patterns in organizations have influenced depressive symptoms within individuals, (2) vice versa, (3) both, or (4) there is a confounding factor that affects both depressive symptoms within individuals and interaction patterns. Although specific causality cannot be established in this study, all possible causal interpretations support collective influences of depression within the organization and suggest that depression in the workplace needs to be dealt with from the perspective of the organization rather than the individual. The clustering coefficient of an interaction network is significant in that it is an observable indicator related to depression within the organization. Future studies could investigate the causality of this link through interventions or longitudinal studies.

In summary, our analysis of comprehensive observations in workplaces involving a variety of occupations shows that the density of face-to-face interactions between surrounding people has a negative correlation with depressive symptoms. This relationship is a group-scale phenomenon that can be confirmed at the group unit level, and it is a phenomenon in which active interaction (i.e., abundant body movements) contributes significantly. Our findings provide a quantitative and collective perspective on taking a systematic approach to depression. In particularly, at this point in time when interactions at workplaces are replaced by interactions in virtual environments and the ways of interaction among workers are changing^68^ due to COVID-19, our findings shed light on the importance of the role of organizational interaction channels in the mental health of employees.

## Methods

### Description of data

A dataset of 470 employees from 10 organizations of Japanese companies collected in 2009 by the ‘Hitachi World Signal Center’ was used for this study. Target organizations included a variety of occupations (financial system, public system, call centre, IT service, personnel application, industrial equipment, medical equipment and insurance), and in all organizations employee interactions were observed for more than 5 days (MEAN = 11.5, SD = 7.2). All workplaces and employees in the dataset are anonymized.

The interactions between employees were measured through a name-tag-type wearable device^33^, which has contributed to the understanding of the collective behaviour of group members^29,46,47,69–72^.

Face-to-face interaction data were detected through an infra-red signal exchanged between the devices of the interacting individuals. The device's built-in infrared modules can detect each other within a distance of 2 m and within a 120° circular sector in front of the device, and communicate the device owner’s ID.

Body movement was measured from the zero-cross count of the acceleration value near the chest. The device's built-in 3-axis accelerometer measures the change in acceleration occurring near the chest with a resolution of 50 Hz, and the zero-cross count of the Euclidean norm of 3-axis acceleration is defined as body movement. Therefore, the measured body movement is closer to the measurement of oscillation, not just the intensity of physical activity, and reflects the people’s activities such as running (over 4 Hz), excited discussion or rushed walking (3–4 Hz), walking or dynamic gesture (2–3 Hz), talking or typing (1–2 Hz), web browsing or listening (0–1 Hz), or sleeping or thinking without movement (0 Hz)^71^.

For comfortable wear for long periods of time, the wearable device is designed in a compact size (73 × 98 × 9 mm, 62 g). We used interaction data with a resolution of 1 min, after processing to reduce power consumption and memory usage^33,71^ (about 24 h without charging).

Our criteria for data pre-processing were as follows. First, we found that there were many short empty intervals between interactions (Supplementary method 1-Fig. S4a, b). These empty intervals may be caused by misalignment between the infrared sensors of the wearable sensors, or may actually be caused by intermittent interaction. However, we judged that it is more reasonable to regard the interactions that occurred over such a short interval as a continuous interaction rather than interactions in a completely new context. So, we set the empty interval criterion to be less than 5 min in this study for reasonable counting of interactions. (We confirmed that the key findings in this study are robust regardless of specific threshold of ‘5 min’ in Supplementary method 1). In addition, since analysis using insufficient wearable device data was unreliable, we targeted 449 employees, excluding 21 employees who did not meet our criteria. These employees had a record of wearable device use of less than 5 h or face-to-face interaction time of less than 1 h.

During the measurement period, when participants arrive at the workplace, they put on their wearable devices and work as usual. All employees of the target organizations were informed of the purpose of the experiment and the use of the data, and the experiment was conducted with those who provided a consent form in accordance with the relevant guidelines and regulations. Only a few people in each organization did not agree to participate. We confirm that informed consent was obtained from all subjects. This experiment was reviewed and approved by Hitachi, Ltd., Research & Development Group.

### Depressive symptoms

Depressive symptoms of individuals were assessed by CES-D (Center for Epidemiologic Studies Depression) which is a self-report depression scale for research in the general population^34^. The CES-D has 20 questions, including 16 negative items such as “I felt depressed”, “People were unfriendly” and 4 positive items such as “I was happy”, “I felt hopeful about the future”. Each item is scored from 0 to 3 according to the number of corresponding days in the past week. The total score ranges from 0 to 60, with a higher value indicating depressive status.

### Social network characteristics

**Figure 5:**
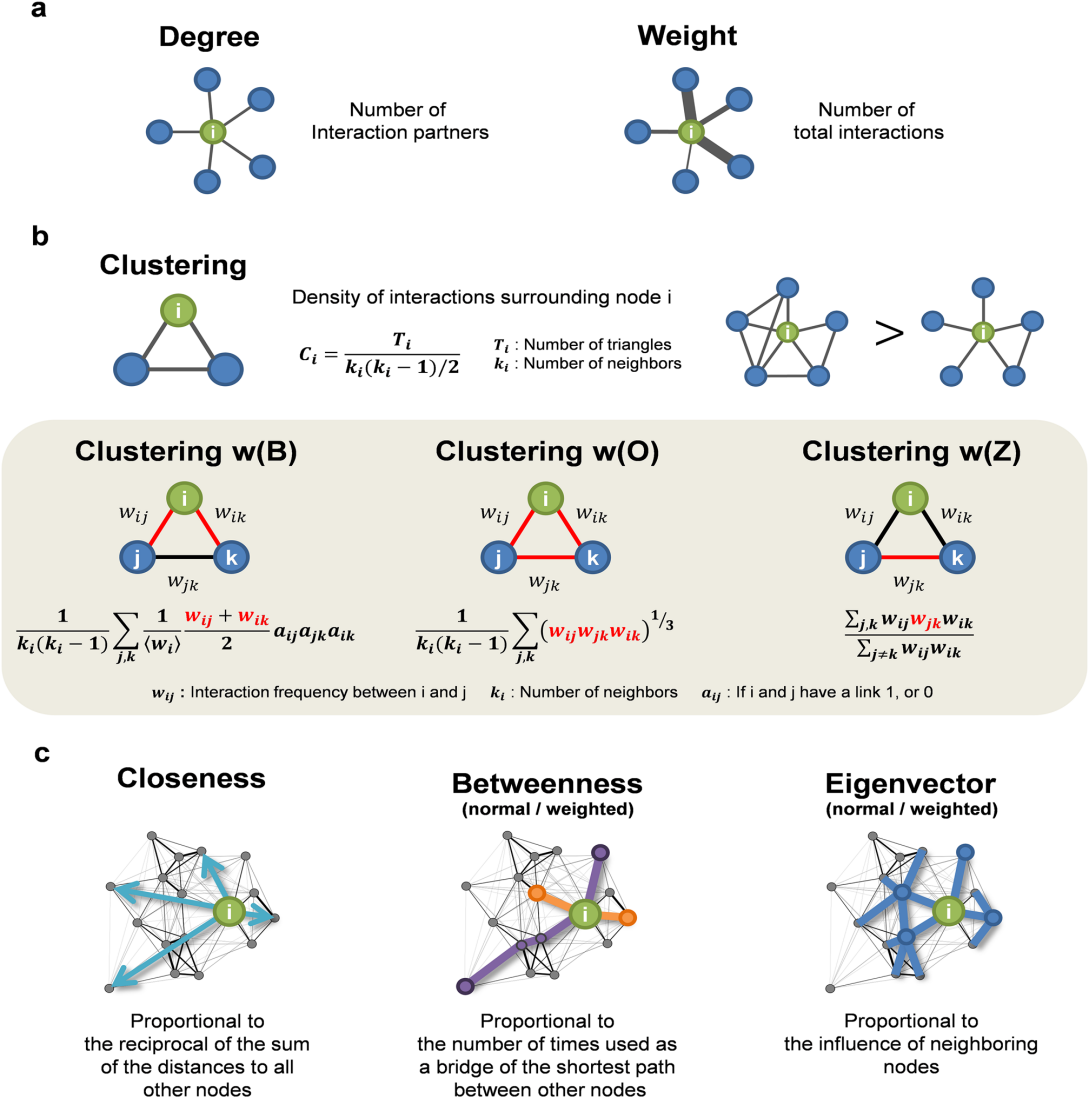
Social network characteristics used in analysis. (**a**) At the individual scale, the number of interaction partners (*Degree*) and the number of total interactions of individual (*Weight*) are used. (**b**), At the surrounding scale, *Clustering*, which quantifies the density of surrounding interactions with the number of triangular structures, and *Clustering w(B)*, *Clustering w(O)*, and *Clustering w(Z)*, which consider the weight of each link of a triangle, are used. (**c**) At the organizational-scale, *Closeness* centrality, which quantifies the proximity to other nodes, *Betweenness* centrality, which quantifies the role of a bridge connecting the shortest path between other nodes, and *Eigenvector* centrality, which quantifies the influence of neighbouring nodes, are used. For a detailed description of each metric, see “Methods” section.

Social networks can be characterized at different scales, based on the scope of interactions. We used 11 social network characteristics defined at individual, surrounding, and organizational scales (Fig. 5). Details for each metric are as follows:

#### Individual scale

Social network characteristics at the individual level are characteristics that can be defined only with individual interaction data. *Degree* refers to the number of interaction partners. *Weight* refers to the average number of interactions per day (i.e., total number of interactions divided by the number of days of measurement).

#### Surrounding scale

In the surrounding level, individuals as well as the interactions between people around them are included in the features. *Clustering* is quantified by the clustering coefficient which indicates the density of interactions around an individual. The clustering coefficient $C_{i}$ of an individual $i$ is defined as a value obtained by dividing the interaction relationship between people around $i$ by the maximum possible value^73^.

$$Clustering_{i} = \frac{{T_{i} }}{{k_{i} \left( {k_{i} - 1} \right)/2}}$$

where $T_{i}$ refers to the number of triangular structures that occur when people around $i$ interact with each other, and $k_{i}$ refers to the number of neighbors of $i$.

In addition, we also introduced three types of weighted clustering coefficients considering the weight of interaction. As for the weight of interactions between employees, the average daily frequency was used as in the individual scope. *Clustering w(B)*, devised by Barrat et al.^74^ imposes a higher weight when sharing a large number of neighbours with people who frequently interact with.

$$Clustering \,w(B)_{i} = \frac{1}{{k_{i} \left( {k_{i} - 1} \right)}}\mathop \sum \limits_{j, k} \frac{1}{{\left\langle {w_{i} } \right\rangle }}\frac{{w_{ij} + w_{ik} }}{2}a_{ij} a_{jk} a_{ik}$$

where $w_{ij}$ refers to the interaction frequency between *i* and *j*, and $a_{ij}$ is 1 when $i$ and $j$ are connected and 0 when not. $\left\langle {w_{i} } \right\rangle$ refers the expected interaction frequency of *i*. *Clustering w(O)*, devised by *Onnela *et al^75^*.* evaluates the strength of the relationship between three people using the geometric mean of their interaction frequency.

$$Clustering \,w(O)_{i} = \frac{1}{{k_{i} \left( {k_{i} - 1} \right)}}\mathop \sum \limits_{j, k} \left( {w_{ij} w_{jk} w_{ik} } \right)^{{{\raise0.7ex\hbox{$1$} \!\mathord{\left/ {\vphantom {1 3}}\right.\kern-\nulldelimiterspace} \!\lower0.7ex\hbox{$3$}}}}$$

The original definition uses $\hat{w}_{ij}$ normalized by the highest $w$ among all link weights instead of $w_{ij}$ to allow the range of coefficients to be defined between 0 and 1. However, since we were only interested in the magnitude of the values, not the coefficient itself, so we did not normalize $w_{ij}$ to apply the same criteria to various organizations. Finally, *Clustering w(Z)*, devised by *Zhang *et al*.*, has the following definitions^76^.

$$Clustering\, w(Z)_{i} = \frac{{\mathop \sum \nolimits_{j, k} w_{ij} w_{jk} w_{ik} }}{{\mathop \sum \nolimits_{j \ne k} w_{ij} w_{ik} }}$$

The original definition uses $\hat{w}_{ij}$ normalized by the highest $w$ among all link weights instead of $w_{ij}$, but in our study we used just $w_{ij}$ for the same reason as *Clustering w(O)*. Zhang et al.^77^ method is a kind of weighted mean for indirect interaction frequency $w_{jk}$ weighted by $w_{ij} w_{ik}$. Therefore, it is relatively insensitive to the weight of direct interaction compared to *Clustering w(O)*, and is more dependent on indirect interaction frequency compared with *Clustering w(B)*^77^.

#### Organizational scale

At the organization level, the interactions among all connected people within an organization are considered in the definition of centrality. *Closeness*^78,79^ centrality evaluates the importance according to distance from other people.

$$Closeness_{i} = \frac{n - 1}{{\mathop \sum \nolimits_{j} d\left( {j, i} \right)}}$$

where $d\left( {j, i} \right)$ refers to the length of the shortest path between $i$ and $j$, and $n$ refers to the number of people in the interaction network. If the distance from all other people is 1, *Closeness* has the largest value of 1 and has a value close to 0 as the distance increases. *Betweenness*^80^ centrality evaluates the importance, which increases as an individual is located on the shortest path between others.

$$Betweenness_{i} = \frac{2}{{\left( {n - 1} \right)\left( {n - 2} \right)}}\mathop \sum \limits_{j, k} \frac{{\sigma_{jk} \left( i \right)}}{{\sigma_{jk} }}$$

where $\sigma_{jk}$ refers to the total number of shortest paths from $j$ to $k$, and $\sigma_{jk} \left( i \right)$ refers to the number of paths passing through $i$ among the number of $\sigma_{jk}$. $2/\left( {n - 1} \right)\left( {n - 1} \right)$ is a regularization term that adjusts to having a value of 1 when centrality is highest. The shortest path is searched through the Dijkstra’s algorithm, and in *Betweenness w*, the Dijkstra’s algorithm for a weighted network is used. *Eigenvector*^81^ centrality evaluates a person’s influence based on the influence of that person’s neighbours.

$$Eigenvector_{i} = \frac{1}{\lambda }\mathop \sum \limits_{j} a_{ij} Eigenvector_{j}$$

where $a_{ij}$ is 1 when $i$ and $j$ are connected and 0 when not, and ${\uplambda }$ refers to the Euclidean normalization term for eigenvector. In the case of *Eigenvector* w, the interaction frequency $w_{ij}$ is used instead of $a_{ij}$.

### Community detection

For detecting community structures, we used the Louvain algorithm^36^, one of the widely used community detection algorithms based on modularity^35,82^. Modularity has a range from − 1 to 1, and a positive value means that the number of internal edges of a given community structure is higher than the expected value when the edges are randomly distributed while preserving the number of edges of each node. Since the face-to-face interaction network can use the interaction frequency as weight information for edge, we used modularity (Q) for a weighted network^35^.

$$Q = \frac{1}{2W}\mathop \sum \limits_{ij} \left( {w_{ij} - \frac{{s_{i} s_{j} }}{2W}} \right)\delta \left( {c_{i} , c_{j} } \right)$$

here $$w_{ij}$$ is the weight between node $$i$$ and $$j$$, $$W$$$$\left( { = \sum\nolimits_{i,j} {w_{ij} /2} } \right)$$ is the sum of weights of all edges, $$s_{i}$$$$\left( { = \sum\nolimits_{i}^{{}} {w_{ij} } } \right)$$ is the sum of weights of node $$i$$, and $${\updelta }\left( {c_{i} , c_{i} } \right)$$ is 1 if nodes $$i$$ and $$j$$ belong to the same community (otherwise 0). In the equation, the $$s_{i} s_{j} /2W$$ term is the expected weight when links are randomly distributed while maintaining the $$s_{i}$$ of each node, and the values obtained by subtracting the expected weight from observed weight $$\left( {w_{ij} } \right)$$ are reflected in modularity.

At the first step of the Louvain algorithm, all nodes start with separate communities, and at the next step one node’s community is relocated to the adjacent community, which gets higher modularity before relocating. This operation is repeated until there is no further change. After this operation, the community is replaced by a single node for which internal edges within communities are represented by self-loop edges. The above operations are repeated until there is no further increase in modularity. The Louvain algorithm may show slightly different detection results depending on the order in which nodes are relocated to the communities, but the modularity of the results is not significantly affected^36^. Therefore, we performed 5000 community detections by randomly mixing the order (Fig. 3c).

In addition, to construct community structures of appropriate size that are modularized while having a high share of the clustering coefficient of members, which enables community detection, we adjusted the power of the weight of each link to its expected value, and the weight of link to the power of 1/4 was used for community detection (Supplementary method 2). As a result, we constructed 34 communities from 10 organizations, and the average number of community members was 13.21 [SD = 6.69] (The number of 34 communities is a result of one sample; the average number of communities of the 5000 samples was 33.46 [SD = 0.69]).

## Supplementary Information

Supplementary Information.

## Data Availability

The data that support the findings of this study are available from the author upon request.

## Code availability

The Python code that supports the findings of this study is available from the author upon request.
