# Supplementary material for: Universal association between depressive symptoms and social-network structures in the workplace
Source: Sci Rep. 2022 Jun 17;12:10170. doi: 10.1038/s41598-022-14366-9 (PMC9205889; doi:10.1038/s41598-022-14366-9)
Supplement: Supplementary file 1 — Supplementary Information. [file 41598_2022_14366_MOESM1_ESM.pdf]

Supplementary information

Universal association between depressive symptoms and social-network structures in the workplace

Jong-Hyeok Lee[1*], Nobuo Sato[2], Kazuo Yano[1, 2], Yoshihiro Miyake[1]

[1]Department of Computer Science, Tokyo Institute of Technology, Yokohama, Kanagawa 226-8502, Japan

[2]Happiness Planet, Ltd., Kokubunji, Tokyo 185-8601, Japan

[*]Corresponding author: lee.j.aw@m.titech.ac.jp

Supplementary note 1 – Correlation between social network characteristics and depression scale in the community unit.

Fig. S1 shows the relationship between social network characteristics and depression scale in the community unit. The method used for the analysis is the same as Fig. 3c. In addition to *Clustering* and *Clustering w(B)*, the correlation observed in *Eigen*, *Eigen w*, and *Closeness* was stronger than that of randomly constructed community (Blue) and randomly constructed community within each organization (Green). *Clustering* and *Clustering w(B)* are partly related to *Eigen*, *Eigen w*, and *Closeness* in that highly clustering structure reduces the length of paths to other nodes and shares influence with each other. However, these indicators (*Eigen*, *Eigen w*, and *Closeness)* are difficult to interpret by themselves compared to *Clustering* and *Clustering w(B)* in that they are not valid for individual unit as shown in Fig. 2a, and their correlation with depression scale is weaker than that of *Clustering* and *Clustering w(B).* In addition, while these indicators are useful for relative comparison within a specific network, there is a disadvantage in that it is difficult to directly compare indices calculated in different networks (especially networks of very different sizes).

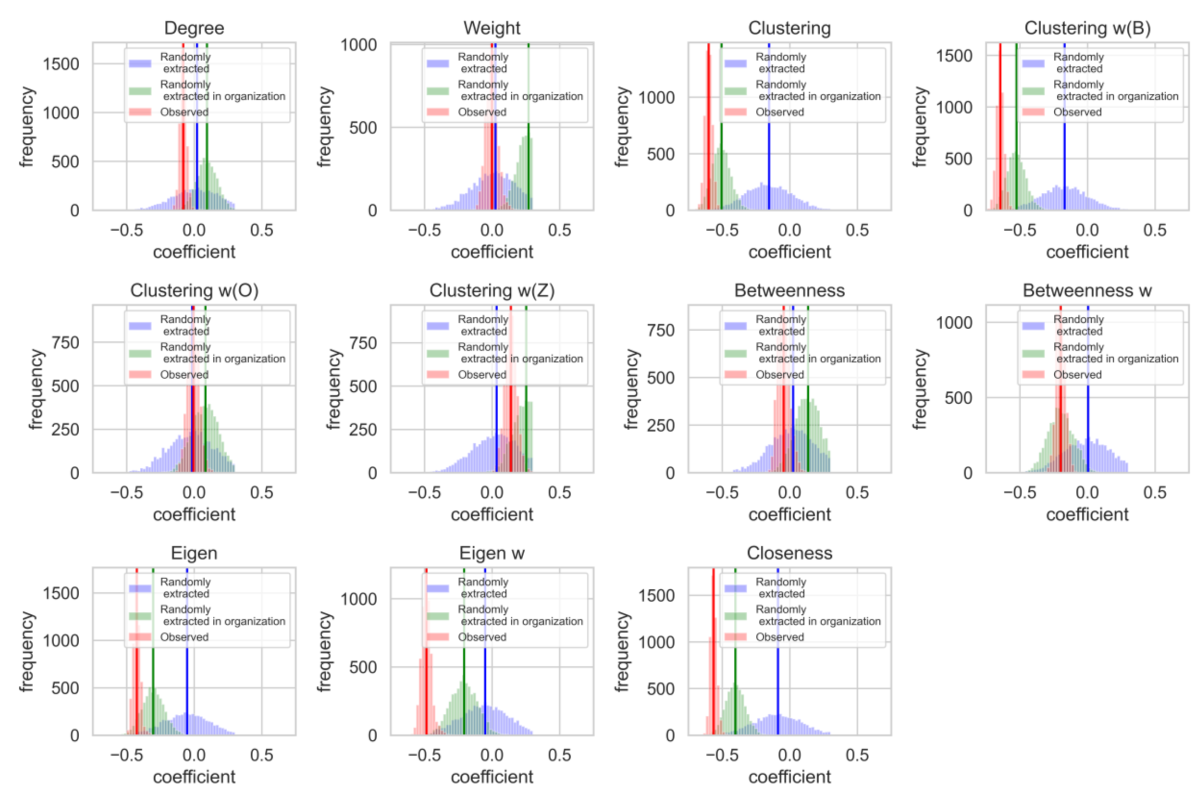

Fig. S1| The distributions of Spearman's correlation coefficient between the average social network characteristics and the average depression scale of each communities. Each distribution represents the Spearman's correlation coefficient of the community structures observed by the community detection algorithm (Red), randomly constructed (Blue), and randomly constructed within each organization (Green), and consisted of 5000 sampling results. (The method used for the analysis is the same as Fig. 3c)

Supplementary note 2 – Observation period.

To investigate the effect of observation period on the correlation between the *Clustering w(B)* and depression scale, we composed the interaction data of N consecutive days of each organization into one network, and investigated the correlation between the *Clustering w(B)* and the depression scale observed in each network (Fig. S2a). Fig. S2b shows the change in the Pearson's correlations of each sample according to the number of consecutive days (N). As N increases, the correlation clearly shows a negative trend, and become significantly lower than 0 over 3 consecutive days (N=3; 95% CI -0.065 to -0.0035). As a result of the same analysis for each organization (Fig. S2c), significant negative trends are observed in 8 out of 10 organizations from observations over 4 days, and become stronger as the observation period increased. These results show that the correlation between *Clustering w(B)* and depression scale reflects the characteristics of chronic interaction between members at the workplace.

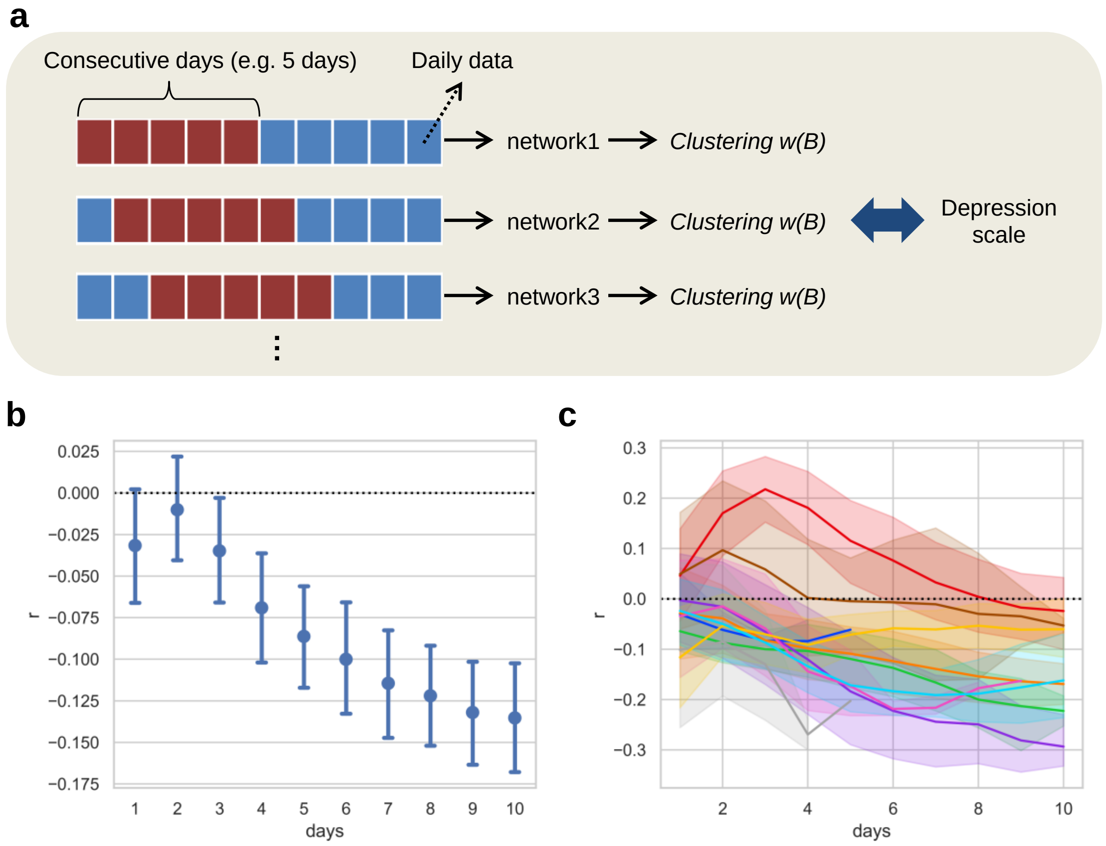

Fig. S2| Correlation between Clustering w(B) and depression scale according to the observation period. a, We constructed the interaction data of N consecutive days into one network, and investigate the Pearson's correlation between *Clustering w(B)* and depression scale of each network. **b, c,** The correlations in the constructed networks according to observation period. Each bar shows 95% confidence interval computed through 5000 bootstrap iterations (**b** represents the results in all generated networks, and **c** represents the results divided by organization). To reduce the noise of *Clustering w(B)* due to short observation period, we only targeted employees who interacted with more than two people.

Supplementary note 3 – Correlation between social network characteristics and specific depressive symptoms.

Fig. S3a shows the Spearman correlation matrix of each item of CES-D and social network characteristics, and Fig. S3b shows a correlation network composed of only relationships where the significance level of each correlation is $p < 0.1$. We note that we do not have CES-D details of all subjects, and only 371 subjects out of 449 were used for this analysis. CES-D consists of 16 negative items and 4 positive items (4, 8, 12, 16), each ranging from 0 to 3 points (the score of the positive term is reversed). And, it is known that CES-D items belong to four factor structures[1]: depressive affect (3, 6, 9, 10, 14, 17, 18; blue node in Fig. S3b, S3d), somatic symptoms (1, 2, 5, 7, 11, 13, 20; yellow nodes), interpersonal problems (15, 19; purple nodes), and positive affect (4, 8, 12, 16; green nodes). Fig. S3c shows the Spearman correlation matrix for CES-D factors (sum of items for each factor) and social network characteristics. And Fig S3d was made in the same way as S3b.

First, from the results of Fig. S3a and S3c, *Clustering w(B)* and *Clustering*, which showed the strongest negative correlation in the total score, showed a high overall negative correlation even when looking at each items. Interestingly, it can be seen that there is a relatively strong correlation with the items corresponding to somatic symptoms: e.g. 7 – I felt that everything I did was an effort, 11 – My sleep was restless, 13 – I talked less than usual. 20 – I could not “get going”. Another interesting point is that *Clustering w(O)* and *Clustering w(Z)*, which weight the frequency of indirect interaction (interactions between people around the individual) more strongly than *Clustering* and *Clustering w(B)*, have a relatively strong negative correlation with interpersonal problems (15 - People were unfriendly, 19 - I felt that people disliked me), which suggests that the relationships with symptoms are slightly different among the clustering indicators.

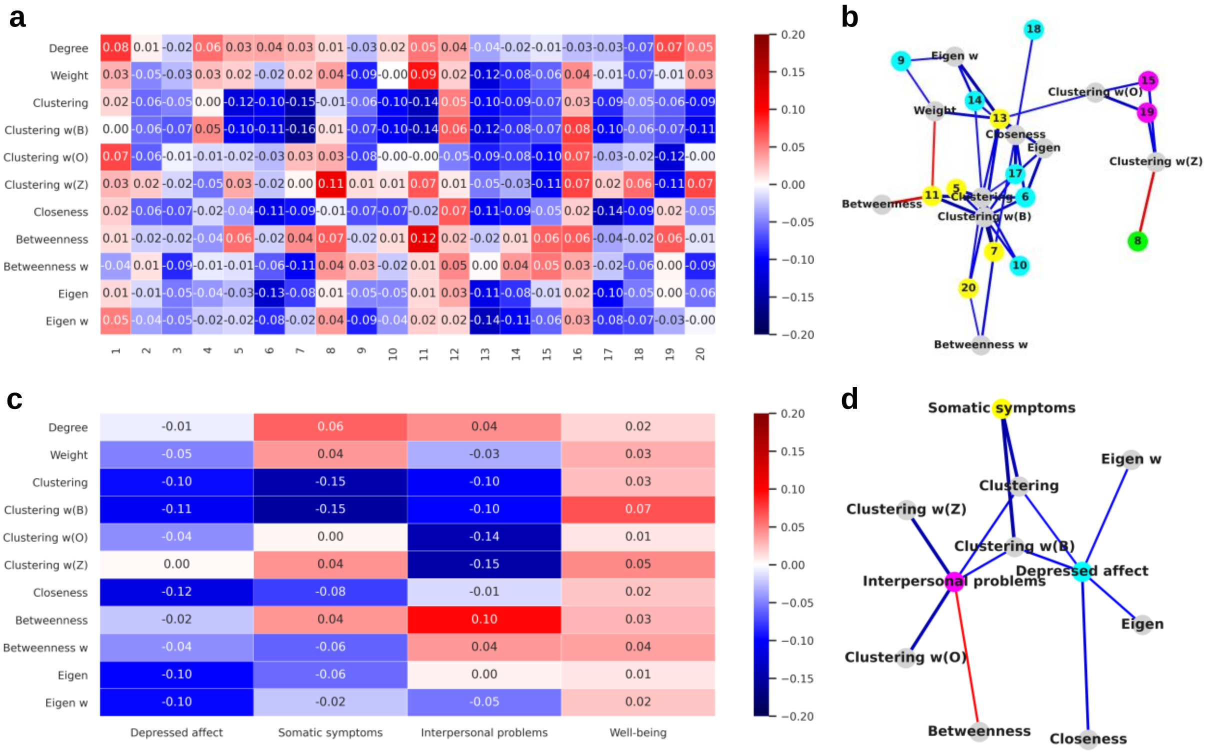

Fig. S3| The Spearman's correlation coefficient between the social network characteristics and specific depressive symptoms. a, Correlation matrix of each item in CES-D and social network characteristics. **b,** A network composed of a relationship of p < 0.1 in the result of **a**. The colour of the node indicates the node's properties: Grey – social network characteristics, yellow – somatic symptoms, blue – depressive affect, purple – depressive affect, green – positive affect. The colour of the link is the same as the colour of the correlation coefficient of **a**, and the width of the link is proportional to the absolute value of the correlation coefficient. **c,** Correlation matrix of sum of items of 4 factors of CES-D and social network characteristics. **d,** A network composed of a relationship of p < 0.1 in the result of **c**.

Supplementary method 1 – the criterion for empty intervals between interactions.

We quantify the weight of the relationship as the number of interactions. However, when we actually looked at the interactions (Fig. S4a), we found that short empty intervals frequently appeared between the interactions (Fig. S4b). These empty intervals may be caused by misalignment between the infrared sensors of the wearable sensors, or may actually be caused by intermittent interaction. However, we judged that it is more reasonable to regard the interactions that occurred over such a short interval as a continuous interaction rather than interactions in a completely new context. We set the interval less than 5 minutes as the criterion for imputation, which is not a long time perceptually, and appears with a high frequency in the data. We think that the frequency of interactions is counted more reasonably through this criterion (Fig. S4a), and we confirmed that our results, the correlation between depressive symptoms and Clustering, Clustering w(B), are robust regardless of the specific threshold of '5 minutes' (Fig. S4c).

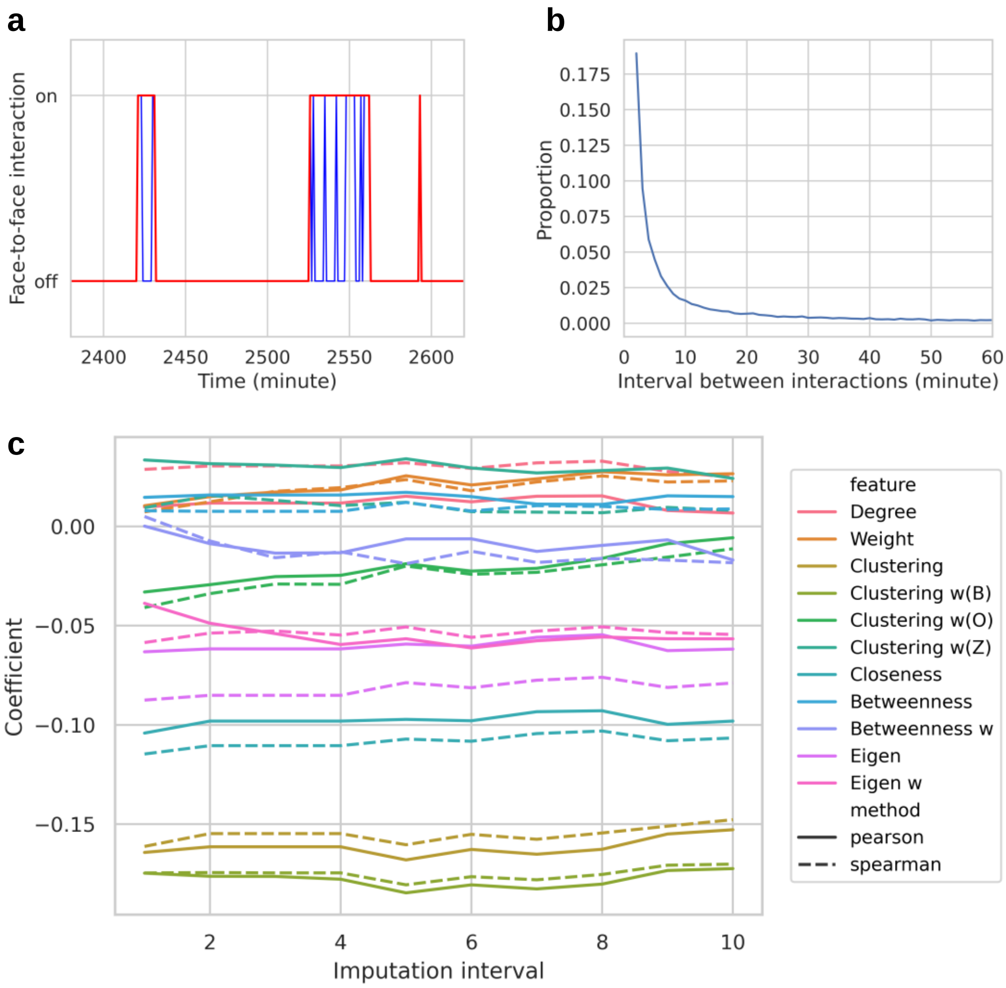

Fig. S4| Imputation of face-to-face interaction data. a, The observed face-to-face interactions between two specific people over a period of about 4 hours. The blue line represents the observed interaction, and the red line represents the corrected interaction. **b,** The proportion distribution of intervals between interactions. It can be seen that short intervals appear with high frequency. **c,** Changes in correlation between depressive symptoms and social network characteristics according to the threshold of the interval used for imputation. It shows that the correlations are robust regardless of the threshold.

Supplementary method 2 – adjustment of resolution in detecting community structures.

Compared with the modularity of unweighted networks, modularity using weight of links can extract community structures with higher resolution and accuracy by using additional information on the frequency of interactions[2]. On the other hand, there is a possibility that many structural features (e.g. clustering coefficient) may be neglected in the extracted community structures, as the connections between people connected by relatively low weight links do not become important.

Therefore we searched for a modular community structure while including a large share of the clustering coefficients of the members by adjusting the power of the weight of links in modularity. The modularity we used for community detection is as follows.

$$\mathrm{Q}_p = \frac{1}{2\widetilde{W}}\sum_{ij}\left(w_{ij}{}^p - \frac{\widetilde{s_i}\widetilde{s_j}}{2\widetilde{W}}\right)\delta(c_i, c_j)$$

Here, p is a parameter for adjusting the power of the weight. Accordingly, $\widetilde{\mathrm{W}} = \sum_{i,j} w_{ij}{}^p/2$ and $\widetilde{s_i} = \sum_i w_{ij}{}^p$ are used instead of W and s_i of ordinary definition of modularity. By adjusting p, communities of various resolutions can be extracted from various power of weighted network including unweighted networks (p=0) and weighted networks (p=1).

Fig. S5 shows the characteristics of community structures extracted through Louvain algorithm according to the adjustment of p. The modularity (Q_w; Fig. S5a) of the original network (G) where p is not applied to weights show that the community structures properly considering the weight of links are detected at a relatively low p (≈0.25). And as p increases, although the resolution of detection is increased (Fig. S5b) but there is no significant increase in the modularity of extracted community structures (Fig. S5a). In addition, the member's proportion of clustering coefficients, degree, and weight within the community decreases (Fig. S5c, d), while the proportion of *Weight* and *Clustering w(B)* within community increase very slightly in the beginning. From these analyses, we determined p as 0.25 to extract the community structures which is sufficiently modularized when considering weight of links and where the proportion of clustering coefficients of members is higher inside than outside.

Fig. S5e shows the Spearman correlation coefficient between average *Clustering w(B)* and depression scale of detected communities according to p. The correlations at p=0.25 are the same as the results in Fig. 3c. The correlations observed in the communities extracted through Louvain algorithm (blue line; Fig. S5e) always showed a stronger negative relationship compared to the correlations observed in the randomly constructed communities regardless of organization (green line; Fig. S5e). Also, it shows a stronger negative relationship compared to the correlation observed in the randomly constructed communities within the same organization (orange line; Fig. S5e) at lower p (<0.8). This result shows the robustness of the correlation at community scale, and suggests that this correlation is evident in a team unit that shared clustering structure internally rather than just a high-resolution team unit entangled with strong weight of links.

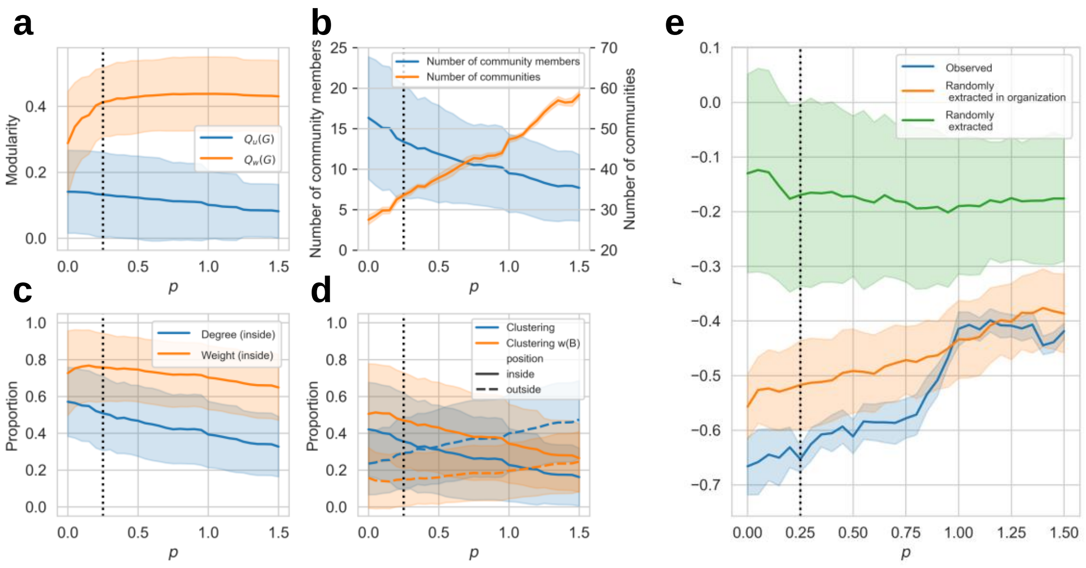

Fig. S5| Characteristics in the community structure detected using modularity that adjusts the power of the link weight. a, Weighted modularity (Q_w) and unweighted modularity (Q_u) of the original network (G). **b,** The number of community members and the total number of communities. **c,** The proportion of *Degree* and *Weight* within the communities in individuals. **d,** The proportion of clustering coefficients within the communities in individuals. *Clustering* and *Clustering w(B)*, which are highly correlated with depression scale, are representatively presented. **e.** The correlations between average *Clustering w(B)* and depression scale of communities (Blue line: the communities detected by Louvain algorithm, Green lines: the communities constructed randomly, Orange lines: the communities constructed randomly within each organization). Random communities are constructed with the same size with the detected communities. Since community extraction in Louvain algorithm is stochastic according to the order of processing nodes, 50 samplings were performed at each p.

References

1. Radloff, L. S. The CES-D scale: A self-report depression scale for research in the general population. *Appl. Psychol. Meas.* **1,** 385–401 (1977).
2. Newman, M. E. J. Analysis of weighted networks. *Phys. Rev. E* **70**, 056131 (2004).
